# Chemoradiotherapy with extended nodal irradiation and/or erlotinib in locally advanced oesophageal squamous cell cancer: long-term update of a randomised phase 3 trial

**DOI:** 10.1038/s41416-020-01054-6

**Published:** 2020-09-22

**Authors:** Congying Xie, Zhao Jing, Honglei Luo, Wei Jiang, Li Ma, Wei Hu, Anping Zheng, Duojie Li, Lingyu Ding, Hongyan Zhang, Conghua Xie, Xilong Lian, Dexi Du, Ming Chen, Xiuhua Bian, Bangxian Tan, Bing Xia, Ruifei Xie, Qing Liu, Lvhua Wang, Shixiu Wu

**Affiliations:** 1 Department of Radiation and Medical Oncology, Second Affiliated Hospital of Wenzhou Medical University, Wenzhou, China; 2 Department of Radiation Oncology, Hangzhou Cancer Hospital, Hangzhou, China; 3 Department of Radiation Oncology, No. 1 People’s Hospital of Huaian, Huaian, China; 4 Department of Radiation Oncology, National Cancer Center/National Clinical Research Center for Cancer/Cancer Hospital & Shenzhen Hospital, Chinese Academy of Medical Sciences and Peking Union Medical College, Shenzhen, 518116 China; 5 Department of Radiation Oncology, Taizhou Hospital of Zhejiang Province, Linhai, China; 6 Department of Radiation Oncology, Anyang Cancer Hospital, Anyang, China; 7 Department of Radiation Oncology, The First Affiliated Hospital of Bengbu Medical College, Bengbu, China; 8 Department of Medical Oncology, Hangzhou Cancer Hospital, Hangzhou, China; 9 Department of Radiation Oncology, Anhui Provincial Hospital, Anhui, China; 10 Department of Radiation Oncology, Zhongnan Hospital of Wuhan University, Wuhan, China; 11 Department of Radiation Oncology, Kaohsiung Medical University Hospital Cancer Centre, Kaohsiung, Taiwan; 12 Department of Radiation Oncology, Central Hospital of Lishui City, Lishui, China; 13 Department of Radiation Oncology, Zhejiang Cancer Hospital, Hangzhou, China; 14 Department of Radiation Oncology, Jiangsu Cancer Hospital, Nanjing, China; 15 Department of Radiation Oncology, Affiliated Hospital of North Sichuan Medical College, Nanchong, China; 16 Department of Biostatistics, Hangzhou Cancer Hospital, Hangzhou, China; 17 Department of Biostatistics, Sun Yat-Sen University Cancer Center, Guangzhou, China

**Keywords:** Oesophageal cancer, Randomized controlled trials

## Abstract

**Background:**

To report the long-term outcomes of a phase III trial designed to test two hypotheses: (1) elective nodal irradiation (ENI) is superior to conventional field irradiation (CFI), and (2) chemoradiotherapy plus erlotinib is superior to chemoradiotherapy in locally advanced oesophageal squamous cell cancer (ESCC).

**Methods:**

Patients with locally advanced ESCC were randomly assigned (1:1:1:1 ratio) to one of the four groups: A: radiotherapy adoption of ENI with two cycles of concurrent TP chemotherapy (paclitaxel and cisplatin) plus erlotinib; B: radiotherapy adoption of ENI with two cycles of concurrent TP; C: radiotherapy adoption of CFI with two cycles of concurrent TP plus erlotinib and D: radiotherapy adoption of CFI with two cycles of concurrent TP. A total of 60 Gy of radiation doses was delivered over 30 fractions. We explored the impact of epidermal growth factor receptor (EGFR) expression on the efficacy of erlotinib plus chemoradiotherapy.

**Results:**

A total of 352 patients (88 assigned to each treatment group) were enrolled. The 5-year survival rates were 44.9%, 34.8%, 33.8% and 19.6% in groups A, B, C and D, respectively (*P* = 0.013). ENI significantly improved OS compared with standard CFI (median, 38.5 vs 22.6 months; HR, 0.74; *P* = 0.018). The addition of erlotinib significantly improved OS (median, 39.4 vs 27.4 months; HR, 0.75; *P* = 0.025). Patients with overexpressing EGFR treated with erlotinib had a better OS and PFS than those without erlotinib.

**Conclusions:**

Concurrent chemoradiotherapy with ENI and/or erlotinib improved long-term survival in locally advanced ESCC.

**Clinical trial registration:**

Trial registration: NCT00686114.

## Background

Oesophageal cancer is one of the most common causes of cancer-related deaths worldwide.^1^ Concurrent chemoradiotherapy (CRT) is deemed the standard of care in patients with locally advanced oesophageal squamous cell cancer (ESCC).^2–4^ Radiotherapy with concurrent chemotherapy of 5-fluorouracil plus cisplatin significantly improved the 5-year overall survival compared with radiotherapy alone in the RTOG 8501 study.^3^ However, the long-term outcome is dismal, particularly when locoregional recurrence is over 50%. To further improve the prognosis of these patients, improved radiotherapy application and more effective drugs are needed.

Our study was designed to investigate the efficacy of elective nodal irradiation (ENI) and/or erlotinib in locally advanced ESCC.^5^ An extensive submucosal lymphatic plexus of the oesophagus leads to a high rate of regional lymph node dissemination in ESCC.^6^ In resectable oesophageal cancer patients, three-field lymphadenectomy was suggested to be superior for thoracic oesophageal cancer by meta-analysis.^7^ Chemoradiotherapy with ENI has been reported to reduce locoregional failure in patients with stage II/III oesophageal cancer in several retrospective studies.^8,9^ In addition, as epidermal growth factor receptor (EGFR) is overexpressed in 30–70% of oesophageal cancers and has been linked to poor prognosis,^10,11^ EGFR inhibitors have been found to reverse the radioresistance of oesophageal cancer cells and have been evaluated in ESCC.^12–14^ The efficacy of EGFR inhibitors was reported to be positively related to EGFR overexpression as well as EGFR gene copy number gain.^15,16^ Xi et al. found that compared to patients with low to moderate EGFR expression, patients with high EGFR expression tended to have a higher response rate using icotinib (0% vs 17.6%, *P* = 0.341) for advanced ESCC.^15^ Similarly, Russell D et al. stated that patients with EGFR FISH-positive advanced oesophageal cancer have an improved OS when treated with gefitinib compared with placebo (*P* = 0.05). However, such a difference was not found in EGFR FISH-negative patients (*P* = 0.46).^16^ We used erlotinib in consideration of its radiosensitisation.^17^ The efficacy and tolerance of concurrent CRT and erlotinib in inoperable ESCC has been reported in a phase II study.^18^ The promising early results of the current study were previously reported.^5^ Chemoradiotherapy with ENI and erlotinib achieved significantly better overall survival and locoregional control than standard chemoradiotherapy in patients with locally advanced ESCC.

This report represents the final update of the trial with respect to treatment efficacy outcomes and toxicities. As required in the protocol, the final survival analysis will be performed once 254 events of death occur. After a minimum follow-up of 5 years for surviving patients and 258 death events, we present an updated analysis. In addition, in an exploratory analysis, we examined the impact of EGFR expression on the efficacy of erlotinib to determine whether EGFR expression can serve as a biomarker for screening patients who are more likely to benefit from this treatment.

## Methods

### Study design and patients

This is a randomised, open-label, phase 3 trial with a 2 × 2 factorial study that was conducted to investigate the efficacy of elective nodal irradiation (ENI) and/or erlotinib in locally advanced oesophageal squamous cell cancer (ESCC). Details of the trial have been described previously.^5^ In brief, patients were 18–75 years of age with histologically proven locally advanced ESCC (stage T1–T4, N0/1, M0–1a according to the 2002 International Union Against Cancer TNM staging system), unsuitable for surgery (comorbidities that preclude surgery or due to patient choice). All patients provided written informed consent, and the study was approved by the institutional review board of each participating centre.

### Randomisation

Eligible patients were randomly assigned (1:1:1:1 ratio) to one of four treatment groups: ENI with two cycles of concurrent TP plus erlotinib (group A); ENI with two cycles of concurrent TP chemotherapy (group B); CFI with two cycles of concurrent TP chemotherapy plus erlotinib (group C) or CFI with two cycles of concurrent TP chemotherapy (group D). Random assignment was performed by the First Hospital of Wenzhou Medical College with a computer-generated random number code. The participants and investigators were not blinded to the treatment allocation.

### Procedures

All patients were treated with a total dose of 60 Gy over 30 fractions in 6 weeks according to routine clinical practice and the treatment guidelines of radiotherapy for Chinese and Japanese oesophageal cancer.^19–22^ The details of the radiotherapy and chemotherapy regimens have been previously reported.^5^ As described previously,^23^ the gross tumour volume (GTV) was defined as the primary tumour, and any enlarged regional lymph nodes were indicated by transoesophageal ultrasound, oesophagram, CT scan and PET/CT (when indicated). The CTV consisted of CTV_1_ and CTV_2_. For patients receiving ENI, the initial CTV1 included the whole oesophagus plus regional lymph nodes. According to the location of the tumour, the regional lymph nodes were prophylactically irradiated. For patients receiving CFI, CTV_1_ was defined as the primary tumour plus the superior and inferior 4-cm margins, radial 1-cm margin and enlarged lymph nodes. After 40 Gy of radiotherapy, CTV_2_ (boost CTV) was defined as GTV plus the superior and inferior 2-cm margins and radial 1-cm margins. Initially, 40 Gy was given to CTV_1_, and a boost dose of 20 Gy was then delivered to CTV_2_. Chemotherapy was prescribed as intravenous paclitaxel (135 mg/m^2^, day 1) and cisplatin (20 mg/m^2^, days 1–3) every 4 weeks for two cycles during radiotherapy. Erlotinib was given (150 mg per day, orally) during chemoradiotherapy.

### Immunohistochemical analysis

Levels of EGFR expression were assessed immunohistochemically. All tissue samples were immersed in 4% paraformaldehyde, embedded in paraffin, and sectioned at a thickness of 8 µm. The sections were probed first with anti-EGFR (1:100, rat polyclonal; Santa Cruz) antibody overnight at 4 °C and then with secondary antibodies for 1 h at room temperature. The evaluation of the immunohistochemical staining was performed independently by two pathologists through light microscopic observation and without knowledge of the clinical data of each patient. The intensity of EGFR expression was defined according to a previously described scoring system^24^: 0: no staining of the cell membrane or staining of the membrane in 10% or less of the tumour cells; 1+: weak and partial staining of the membrane in more than 10% of tumour cells; 2+: moderate and complete staining of the membrane in more than 10% of tumour cells; 3+: strong and complete staining of the membrane in more than 10% of tumour cells. A score of 0–1+ was defined as without EGFR expression, while a score of 2+ to 3+ was defined as EGFR overexpression.

### Outcomes

The primary endpoint was overall survival (OS). The secondary endpoints included progression-free survival (PFS) and treatment toxicity. Response to treatment was evaluated using the Response Evaluation Criteria in Solid Tumors (RECIST 1.1). Adverse events were graded according to the National Cancer Institute Common Terminology Criteria for Adverse Events (version 3.0).

### Statistical analysis

We assumed that the 2-year survival rate can increase from 35 to 45% with the addition of ENI or erlotinib to concurrent chemoradiotherapy for oesophageal cancer. With a minimum 2-year follow-up and 5% annual dropout, the trial needs to recruit 344 patients (86 per group) to have a power of 85% to detect an improvement in 2-year survival.

Efficacy analyses were performed on all randomised patients (intention-to-treat [ITT] population), and safety analyses were performed on the per-protocol population. The per-protocol population was defined as receiving at least one cycle of chemotherapy and a radiation dose of more than 50 Gy. Patients allocated to erlotinib should receive at least 5 weeks of erlotinib treatment.

All time-related endpoints were measured from the date of treatment initiation. Overall survival and 5-year survival rates and their 95% confidence intervals (CIs) were estimated by the Kaplan–Meier method. Survival curves were compared with the log-rank test. The 2 × 2 factorial trial adopted the method by Crowley and Hoering.^25^ If the proportional hazard test was not of statistical significance by the method of PM Grambsch & TM Themeau and the proportional hazard assumption holds, the Cox proportional hazard model was used to analyse the survival effect in this 2 × 2 factorial design.^26^ The interaction between ENI and erlotinib was tested first. If no significant interaction was detected, we performed the main effect analysis (ENI vs CFI or erlotinib vs non-erlotinib). If the interaction between ENI and erlotinib was significant, we considered pairwise comparisons between ENI + erlotinib, ENI without erlotinib, CFI + erlotinib and CFI only. Hazard ratios (HRs) with 95% CIs were calculated using a Cox regression model. We performed a multivariable analysis with the Cox regression model of predefined baseline characteristics to examine the effect of treatment after adjustment for other statistically significant prognostic factors (sex, tumour location, T stage, clinical stage and ECOG performance status). A post-hoc analysis of OS outcomes according to EGFR expression was conducted as an exploratory analysis. Chi-square tests and ANOVA were used to evaluate differences in patient group characteristics and treatment toxicities. We performed the statistical analyses with SAS (version 9.3) and R (version 3.1.3). A *P* value < 0.05 is considered statistically significant, and all reported *P* values are two-sided.

This trial is registered with ClinicalTrials.gov, number NCT00686114.

## Results

**Fig. 1:**
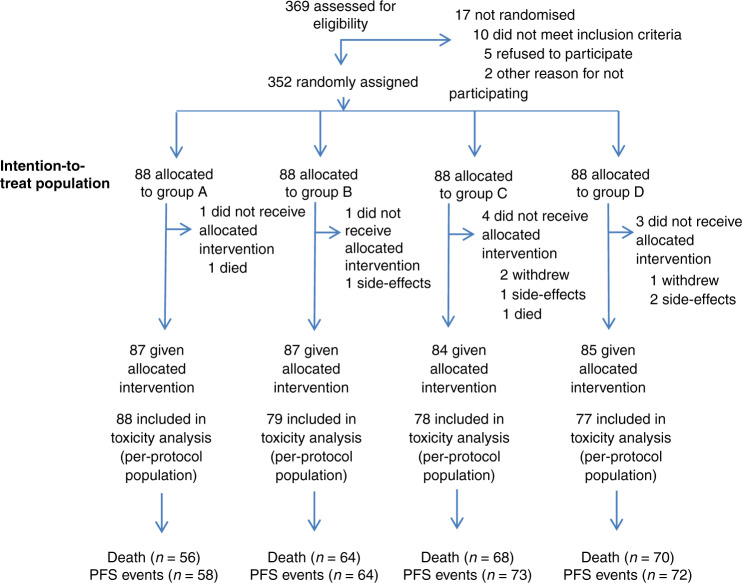
Flow diagram. N number of patients. Group A: chemotherapy/elective nodal irradiation + erlotinib; Group B: chemotherapy/elective nodal irradiation; Group C: chemotherapy/conventional field irradiation + erlotinib; Group D: chemotherapy/conventional field irradiation.

A total of 369 patients were recruited for the eligibility assessment, and 352 patients (88 patients each in groups A, B, C and D) were enrolled between December 2007 and June 2015 from 14 institutions in China. The allocation of patients by treatment arm and their outcomes are shown in the CONSORT diagram in Fig. 1. There were no significant differences in baseline characteristics among the four groups (Table 1). The median age was 61 years (range, 35–70 years), 83.5% had T3-4 disease and 57.5% had node-positive cancer.

**Table 1:** Baseline characteristics of randomly assigned patients.

Characteristics	No. (%)	Group A (*n* = 88)	Group B (*n* = 88)	Group C (*n* = 88)	Group D (*n* = 88)	*p*
		*N* (%)	*N* (%)	*N* (%)	*N* (%)	
Sex						
Male	288 (81.8)	72 (81.8)	76 (86.4)	68 (77.3)	72 (81.8)	0.486
Female	64 (18.2)	16 (18.2)	12 (13.6)	20 (22.7)	16 (18.2)	
Age (years)						
Median	61	61	61	61	62	0.770
Range	35–70	35–70	40–68	40–70	41–70	
Tumour length (cm)						
Median	5.3	5	5.3	6	5.6	0.130
Range	1.2–15	1.5–14	2–15	1.2–11.2	2.9–12	
T stage						
T1	11 (3.1)	1 (1.1)	2 (2.3)	6 (6.8)	2 (2.3)	0.067
T2	47 (13.4)	19 (21.6)	10 (11.4)	11 (12.5)	7 (8.0)	
T3	193 (54.8)	48 (54.5)	45 (51.1)	50 (56.8)	50 (56.8)	
T4	101 (28.7)	20 (22.7)	31 (35.2)	21 (23.9)	29 (33.0)	
N stage						
N−	149 (42.3)	39 (44.3)	33 (37.5)	40 (45.5)	37 (42.0)	0.730
N+	203 (57.7)	49 (55.7)	55 (62.5)	48 (54.5)	51 (58.0)	
ECOG PS						
0–1	218 (61.9)	55 (62.5)	54 (61.4)	55 (62.5)	54 (61.4)	0.995
2	134 (38.1)	33 (37.5)	34 (38.6)	33 (37.5)	34 (38.6)	
Tumour location						
Cervical	21 (6.0)	4 (4.5)	6 (1.7)	5 (5.7)	6 (6.8)	0.999
Upper thoracic	103 (29.3)	24 (27.3)	26 (7.4)	27 (30.7)	26 (29.5)	
Middle thoracic	195 (55.4)	51 (58.0)	48 (13.7)	47 (53.4)	49 (55.7)	
Lower thoracic	33 (9.4)	9 (10.2)	8 (2.3)	9 (10.2)	7 (8.0)	
EGFR expression						
Unknow	262 (74.4)	64 (72.7)	65 (73.9)	67 (76.1)	66 (75.0)	0.441
0	16 (4.5)	2 (2.3)	5 (5.7)	2 (2.3)	7 (8.0)	
1+	23 (6.5)	6 (6.8)	8 (9.1)	6 (6.8)	3 (3.4)	
2+	31 (8.8)	9 (10.2)	6 (6.8)	6 (6.8)	10 (11.4)	
3+	20 (5.7)	7 (8.0)	4 (4.5)	7 (8.0)	2 (2.3)	

*N* number of patients, *Group A* chemotherapy/extended nodal irradiation + erlotinib, *Group B* chemotherapy/extended nodal irradiation, *Group C* chemotherapy/conventional field irradiation + erlotinib, *Group D* chemotherapy/conventional field irradiation, *ECOG PS* Eastern Cooperative Oncology Group performance status, *EGFR* epidermal growth factor receptor.

This report updates outcomes through October 2018. The minimum follow-up was 5 years for surviving patients. A total of 267 patients (76% of eligible patients) experienced treatment failure, locoregional only in 147 patients (55.1%), distant only in 101 patients (37.8%) and both locoregional and distant in 19 patients (7.1%). There were 258 deaths (72.3%), which met the demand of the final analysis.

**Fig. 2:**
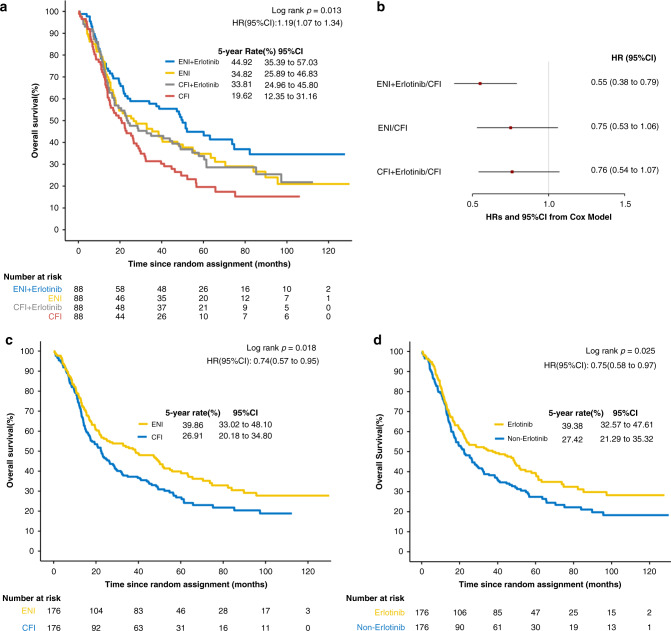
Overall survival in the intention-to-treat population. **a** Kaplan–Meier estimates by treatment group. **b** Estimated hazard ratios (HRs) and 95% CIs from the stratified univariable Cox models in four groups. **c** Kaplan–Meier estimates by radiation type (ENI vs CFI). **d** Kaplan–Meier estimates by erlotinib administration (with erlotinib vs without erlotinib).

The proportional hazard test was not significant (Supplementary Table 1). Among the four groups, there was a significant difference in overall survival (stratified log-rank *P* = 0.013). The 5-year survival rates were 44.9%, 34.8%, 33.8% and 19.6% in groups A, B, C and D, respectively (HR, 1.2; 95% CI: 1.07–1.34; *P* = 0.013, Fig. 2a). Stratified Cox model analysis also showed that patients in the ENI plus erlotinib group (HR, 0.55; *P* < 0.001) had a significantly lower hazard of death than patients in the standard CFI group (Fig. 2b). Univariate analysis by the Cox model showed that sex (*P* = 0.032), T stage (*P* = 0.004) and ECOG performance status (*P* = 0.001) were prognostic factors related to OS. After adjusting for sex, T stage and ECOG performance status, ENI (*P* = 0.018) and erlotinib (*P* = 0.025) were significantly correlated with OS (Supplementary Table 2).

In a stratified Cox model that included the radiation administered (ENI vs CFI), the erlotinib administration (erlotinib vs non-erlotinib), and their interaction, the radiation field-by-erlotinib interaction test was not significant for OS (HR, 0.96; *P* = 0.871) or PFS (HR, 0.97; *P* = 0.908) (Supplementary Tables 3 and 4). The median OS was 38.53 months (95% CI, 22.47–52.07) for patients treated by ENI (groups A and B) and 22.60 months (95% CI, 17.2–28.73) for patients treated with CFI (groups C and D) (Fig. 2c). As in the preliminary report, there remained a significant difference in OS between the ENI groups and the CFI groups (HR, 0.74; 95% CI: 0.57–0.95; log-rank *P* = 0.018). Stratified Cox model analysis also showed that patients in the ENI groups had a significantly lower hazard of death than patients in the CFI groups (HR, 0.69; 95% CI: 0.54–0.89; *P* = 0.005). The median OS was 37.37 months (95% CI: 22.6–51.43) for patients treated with erlotinib (groups A and C) and 22.33 months (95% CI, 17.7–29.9) for patients not treated with erlotinib (groups B and D) (HR, 0.75; 95% CI: 0.58–0.97; log-rank *P* = 0.025; Fig. 2d). Stratified Cox model analysis showed that patients in the erlotinib group (HR, 0.75; 95% CI: 0.58–0.96; *P* = 0.023) had a lower hazard of death than patients in the non-erlotinib group.

**Fig. 3:**
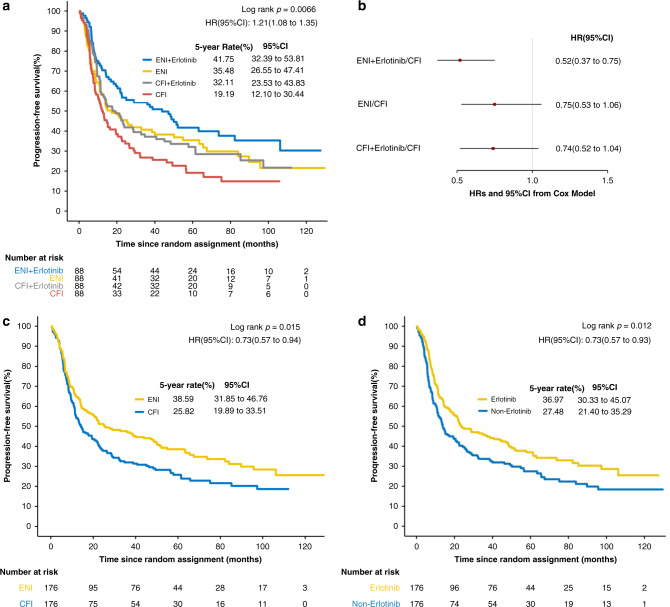
Progression-free survival in the intention-to-treat population. **a** Kaplan–Meier estimates by treatment group. **b** Estimated hazard ratios (HRs) and 95% CIs from the stratified univariable Cox models in four groups. **c** Kaplan–Meier estimates by radiation type (ENI vs CFI). **d** Kaplan–Meier estimates by erlotinib administration (with erlotinib vs without erlotinib).

The PFS curves are shown in Fig. 3a. Among the four groups, there was also a significant difference in PFS (HR, 1.21; 95% CI: 1.08–1.35; stratified log-rank *P* = 0.0066). Stratified Cox model analysis showed that patients in the ENI plus erlotinib group (HR, 0.52; *P* < 0.001) had a significantly lower hazard of disease progression than patients in the standard CFI group (Fig. 3b).

The median PFS was 25.27 months (95% CI: 18.23–49.97) in the ENI groups and 14.20 months (95% CI: 11.37–21.9) in the CFI groups (Fig. 3c). Patients in the ENI group had a significantly longer PFS than patients in the CFI group (HR, 0.73; 95% CI: 0.57–0.94; log-rank *P* = 0.015). The median PFS was 23.33 months (95% CI: 18.23–47.2) in the erlotinib group and 13.80 months (95% CI: 11.0–22.33) in the non-erlotinib group (Fig. 3d). There was a significant difference between the erlotinib and non-erlotinib groups (HR, 0.73; 95% CI: 0.57–0.93; log-rank *P* = 0.012).

Acute adverse events have been presented in earlier analyses.^5^ The most common late adverse event was oesophageal stenosis, which occurred in 19 patients (11.9%) in the ENI group and in 18 patients (13.5%) in the CFI group. Severe late radiation-associated toxicities affecting the skin, lung and heart were rare, with three patients in the ENI group and two in the CFI group suffering from symptomatic cardiac disorders (Table 2).

**Table 2:** Grade 3–4 late adverse events.

	Group A	Group B	Group C	Group D	*p*
	*N* = 88	*N* = 88	*N* = 88	*N* = 88	
Esophageal stenosis	9	10	11	7	0.800
Cardiac disorders	2	1	1	1	0.896

*Group A* chemotherapy/extended nodal irradiation + erlotinib, *Group B* chemotherapy/extended nodal irradiation, *Group C* chemotherapy/conventional field irradiation + erlotinib, *Group D* chemotherapy/conventional field irradiation.

There were four treatment-related deaths: two deaths due to oesophageal perforation (in groups C and D), one death due to pneumonia (group A) and one death due to cachexia (group D).

### EGFR expression and prognosis

**Fig. 4:**
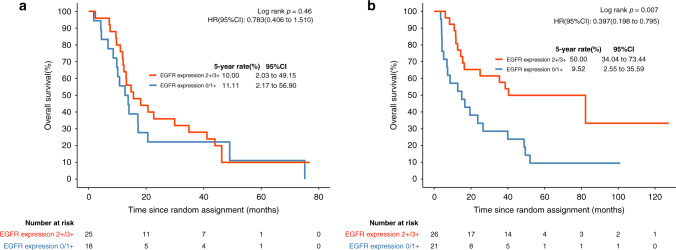
Overall survival stratified by EGFR expression status. **a** Kaplan–Meier estimates in patients without erlotinib treatment by EGFR expression status. **b** Kaplan–Meier estimates in patients treated with erlotinib by EGFR expression status. HR hazard ratio.

EGFR expression was evaluated in patients with sufficient biopsy specimens for immunohistochemical examination. Ninety biopsy specimens from 352 patients were evaluated. Sixteen patients exhibited no detectable EGFR expression, 23 patients showed +1 expression, 31 patients showed +2 expression and the remaining 20 patients showed +3 expression (Table 1). Patients with EGFR expression (+2, +3) had a shorter OS than patients without EGFR expression (0, +1) (median, 21.0 vs 22.2 months; *P* = 0.46, Fig. 4a), consistent with the findings of previous research reports.^27–29^ When treated with erlotinib, patients with EGFR expression (+2, +3) had a significantly longer OS than patients without EGFR expression (0, +1) (median, 46.5 vs 9.5 months; *P* = 0.007, Fig. 4b). The clinical features of the EGFR-tested group and nontested group are shown in Table S5. Table S6 shows the clinical features of patients with and without EGFR expression. OS was worse in those patients who had EGFR IHC performed than in those who did not (*P* = 0.003, 20.3 vs 37.5 months, Fig. S1).

## Discussion

After a minimum follow-up of 5 years for surviving patients, we report the long-term outcomes of a trial that was designed to determine the efficacy of elective nodal irradiation and/or erlotinib in locally advanced ESCC. In this final analysis of the trial, with longer follow-up, the current results are consistent with our early results. The median OS was 49.97, 26.50, 23.83 and 20.87 months in groups A, B, C and D, respectively. The comparison revealed that ENI significantly prolonged OS and PFS compared with CFI in locally advanced ESCC. OS and PFS were significantly prolonged in patients treated with chemoradiation plus erlotinib compared with those treated without erlotinib.

Due to the unsatisfactory outcomes of radiation dose escalation in previous large clinical trials, radical radiation doses of 50–50.4 Gy are widely recommended worldwide.^2,4^ However, owing to the unique high prevalence of ESCC in Asia, a relatively higher dose together with concurrent chemotherapy is commonly used and generally yields favourable outcomes.^22,30^ In our study, a total dose of 60 Gy was prescribed, which was a commonly used dose suggested by the treatment guidelines of China and Japan. To conquer multicentric disease or submucosal skip metastasis due to the rich submucosal lymphatics in the oesophagus, several studies represented by RTOG 85–01 adopted an extended nodal field, but no consensus has been established because of the toxicity and similar survival outcomes compared to involved-field irradiation (IFI).^2,31–35^ Moreover, a large retrospective study showed the superiority of ENI in lymph node-positive oesophageal squamous cell carcinoma patients.^36^ Our study is the first randomised clinical trial to evaluate ENI vs CFI.

The superiority of ENI or erlotinib with chemoradiotherapy is unlikely attributable to underperformance in the standard chemoradiotherapy group (group D). The 5-year survival rate achieved in group D was 19%, which was similar to the rates observed in other studies.^18,19^ This analysis revealed that concurrent chemotherapy with ENI and erlotinib can achieve a 48% reduction in the risk of death. ENI or erlotinib was associated with an ~21% or 14% absolute improvement in OS at 5 years. This finding clearly establishes concurrent chemotherapy with ENI and/or erlotinib as an effective regimen for locally advanced ESCC.

The toxicities were as expected, with no evidence of new safety signals. The incidence of radiation oesophagitis increased in the ENI groups compared with the CFI groups (*P* = 0.027). Nevertheless, oesophageal stenosis was similar between the two radiation types (*P* = 0.8). The incidence of rash increased in the erlotinib groups compared with the non-erlotinib groups (*P* = 0.012). In the present study, the incidence of grade 3–4 late toxicities was similar to that reported in a previous study.^37^ In general, erlotinib and ENI can be safely added to chemoradiotherapy.

EGFR is a transmembrane receptor and one of the members of the ERBB family. It is involved in cancer cell proliferation, the prevention of apoptosis, tumour-induced angiogenesis and tumour progression. Different alterations in EGFR have been identified in oesophageal cancer, including overexpression, copy number gain, etc.^16,38,39^ Previous studies found that EGFR overexpression and EGFR gene copy number gain were associated with poor prognosis.^10,11^ Patients with EGFR overexpression or EGFR gene copy number gain tended to benefit more from EGFR-TKI treatment.^15,16^ In this study, immunohistochemical analysis confirmed that 51 (56.7%) of 90 patients overexpressed EGFR. This finding was consistent with the earlier observation that EGFR was overexpressed in 30–70% of ESCC cases.^40^ The expression of EGFR was reported to be significantly correlated with clinical stage, tumour invasion and poor prognosis.^27–29,41^ Similarly, our study indicated that EGFR expression was associated with T stage and poor prognosis. Radiotherapy with EGFR tyrosine kinase inhibitors has been tested in many trials with encouraging results.^42–44^ Two phase II studies reported the promising efficacy of concurrent radiotherapy with gefitinib or erlotinib in elderly oesophageal cancer patients.^45,46^ EGFR tyrosine kinase inhibitor therapy can reverse poor outcomes in oesophageal cancer.^16,18^ Our results demonstrated that EGFR expression estimated a poor prognosis and could serve as a predictive biomarker for erlotinib administration with CRT. The OS of patients overexpressing EGFR (+2, +3) treated with erlotinib plus chemoradiotherapy was significantly longer than that of patients without EGFR expression (0, +1) (median 46.5 vs 9.5 months). The OS of patients without EGFR expression (0, +1) treated with erlotinib was significantly shorter than that of patients without erlotinib treatment (median 9.5 months vs 22.2 months). The results indicated that EGFR overexpression had potential predictive capability for the outcome of patients treated with erlotinib plus chemoradiotherapy. The use of erlotinib in combination with definitive chemoradiotherapy in locally advanced ESCC patients without EGFR expression may be harmful. We also found that OS was worse in patients who had EGFR IHC performed than in those who did not. This difference is likely due to the chance of biopsy availability. Since EGFR IHC scores were distributed evenly in different trial treatment arms, this result further emphasises the beneficial impact of erlotinib in EGFR IHC-positive patients because the EGFR IHC cohort represents a poorer outcome group in our trial. The negative results of SCOPE1 may be partly due to the absence of incorporating biomarker-driven anti-EGFR therapy with definitive chemoradiotherapy.^47^ In addition, the study enrolled patients with a mixture of squamous cell cancer and adenocarcinoma, which may lead to the benefits of cetuximab in combination with CRT being underestimated. Different RT regimens and chemotherapy regimens between SCOPE1 and our study may also contribute to the contradictory results.

Tumour immunotherapy has gained increasing interest. The role of radiotherapy in promoting inflammation, leading to the infiltration and activation of immune cells, thus enhancing the efficacy of immunotherapy, has been proven.^48,49^ A recent study found that compared with tumour stereotactic radiotherapy, ENI restrained immune infiltration and adversely impacted the efficacy of combined RT and immunotherapy. However, the fractionation used in the study was relatively large (12 Gy).^50^ Whether the conclusion is applicable to conventional radiotherapy and clinical practice remains unknown. Further investigation is warranted.

There were some limitations of our study. As the EGFR IHC test was a post-hoc analysis, many patients did not have sufficient biopsy specimens for immunohistochemical examination. Only 90 (25.6%) patients underwent EGFR evaluation, which may limit the detection efficacy of erlotinib. Although the toxicity of ENI with 60 Gy radiotherapy concurrent with chemotherapy was acceptable, additional investigation is needed to validate the conclusion in 50 Gy radiotherapy. PET scans were not performed in all cases, so occult metastatic disease may contribute to death and weaken the power of the study to assess the benefits of local therapy. However, the randomised design reduced this bias.

In conclusion, our results demonstrated that ENI and/or erlotinib in addition to conventional chemoradiotherapy significantly improved both OS and PFS in patients with locally advanced or medically inoperable ESCC. This regimen represents a substantial improvement in the standard of care for locally advanced ESCC. EGFR overexpression may be a good predictive biomarker for the outcome of patients treated with erlotinib plus chemoradiotherapy.

## Supplementary information

Supplementary file

## Data Availability

All data generated or analysed during this study are available from the corresponding authors upon reasonable request.
