# Supplementary material for: Chemoradiotherapy with extended nodal irradiation and/or erlotinib in locally advanced oesophageal squamous cell cancer: long-term update of a randomised phase 3 trial
Source: Br J Cancer. 2020 Sep 22;123(11):1616–24. doi: 10.1038/s41416-020-01054-6 (PMC7686329; doi:10.1038/s41416-020-01054-6)
Supplement: Supplementary file 1 — Supplementary file [file 41416_2020_1054_MOESM1_ESM.docx]

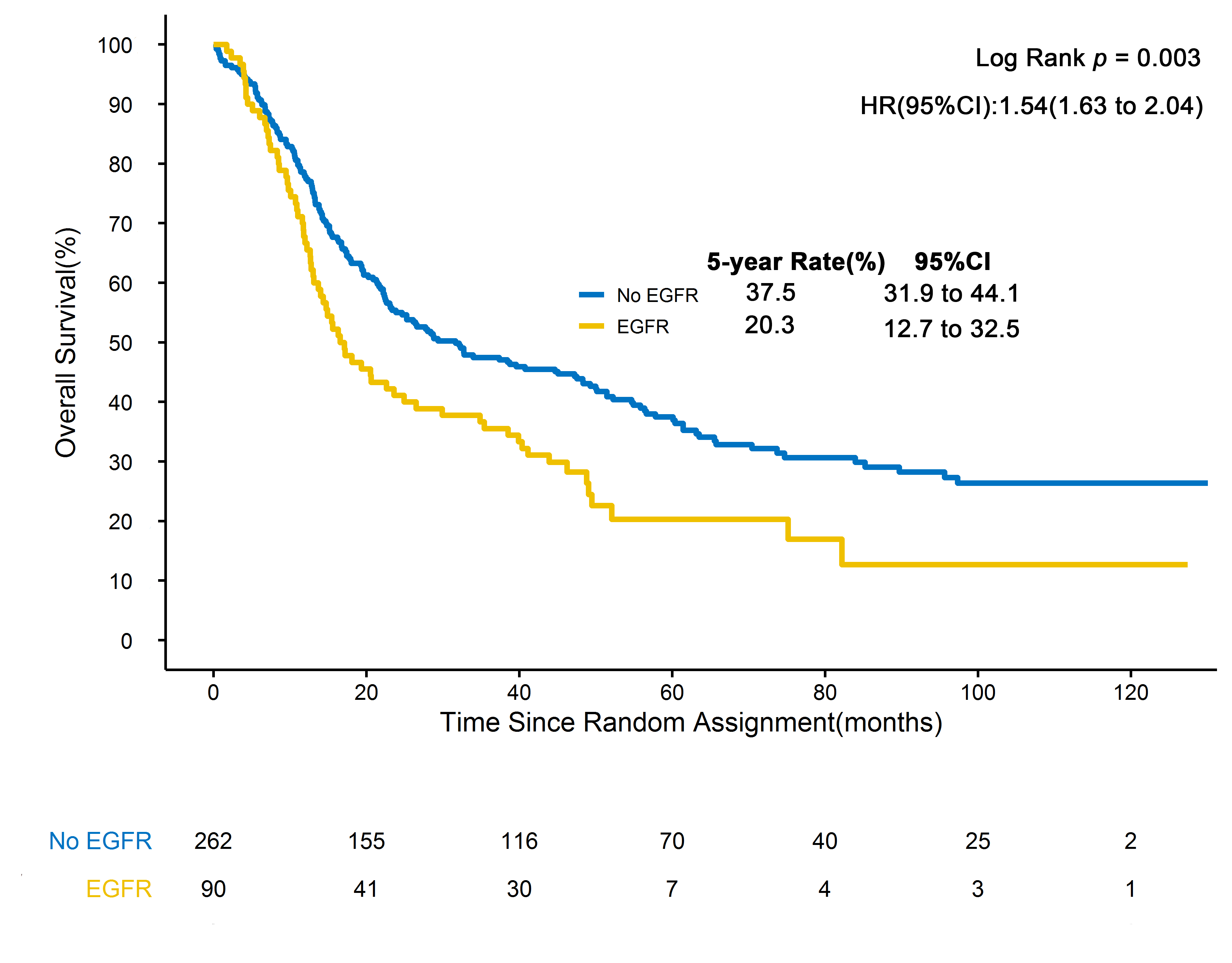


Figure S1. Overall survival for EGFR tested group and non-tested group

Table S1. Proportional hazard Test

| Group | *Ｘ^2^* | *P* |
| --- | --- | --- |
| ENI/CFI, OS | 0.415 | 0.519 |
| ENI/CFI, PFS | 0.514 | 0.473 |
| Erlotinib/Without, OS | 0.331 | 0.565 |
| Erlotinib/Without, PFS | 7.240 | 0.007* |
| group1-4, OS | 0.550 | 0.458 |
| group1-4, PFS | 2.390 | 0.122 |
| Group2-4, OS | 0.758 | 0.384 |
| Group2-4, PFS | 1.950 | 0.163 |
| Group3-4, OS | 0.327 | 0.568 |
| Group3-4, PFS | 2.360 | 0.124 |

ENI, extended nodal irradiation; CFI, conventional field irradiation; OS, overall survival; PFS, progression-free survival.

Table S2. Univariate analysis of prognosis factors related with OS and multivariate analysis for adjusted confounding factors by Cox model

| Effect |  | HR | 95% CI | P value |
| --- | --- | --- | --- | --- |
| Sex |  | 0.68 | 0.48 to 0.97 | 0.032 |
| Age group |  | 0.99 | 0.98 to 1.01 | 0.531 |
| Location | Cervical | 0.70 | 0.43 to 1.14 | 0.151 |
| Ref. Lower | Upper | 0.78 | 0.55 to 1.11 | 0.161 |
|  | Middle | 0.90 | 0.66 to 1.24 | 0.527 |
| T stage |  | 1.29 | 1.08 to 1.54 | 0.004 |
| N stage |  | 0.94 | 0.73 to 1.21 | 0.602 |
| ECOG PS |  | 1.40 | 1.15 to 1.70 | 0.001 |
| ENI/CFI* |  | 0.74 | 0.57 to 0.95 | 0.018 |
| Erlotinib* |  | 0.75 | 0.58 to 0.97 | 0.025 |

OS, overall survival; HR, hazard ratio; CI, confidence interval; ECOG PS, Eastern Cooperative Oncology Group performance status

*Adjusted for sex, location, T stage and ECOG PS

Table S3. Factorial analysis of 2×2 design by Cox model for OS survival

| Effect |  | HR | 95% CI | P value |
| --- | --- | --- | --- | --- |
| CFI |  | 1 |  | 0.015 |
| ENI |  | 0.75 | 0.53 to 1.06 | 0.104 |
| Erl |  | 0.76 | 0.54 to 1.08 | 0.126 |
| ENI+Erl |  | 0.55 | 0.38 to 0.79 | 0.001 |
| Interactive effect | |  |  |  |
| ENI*Erl |  | 0.96 | 0.58 to 1.59 | 0.871 |
| Main effect | |  |  |  |
| ENI |  | 0.75 | 0.53 to 1.06 | 0.104 |
| Erlotinib |  | 0.76 | 0.54 to 1.08 | 0.126 |

Table S4. Factorial analysis of 2×2 design by Cox model for PFS survival

| Effect |  | HR | 95% CI | P value |
| --- | --- | --- | --- | --- |
| CFI |  | 1 |  | 0.007 |
| ENI+Erl |  | 0.53 | 0.37 to 0.76 | 0.001 |
| ENI |  | 0.75 | 0.53 to 1.05 | 0.093 |
| Erl |  | 0.74 | 0.52 to 1.04 | 0.082 |
| Interactive effect | |  |  |  |
| ENI*Erl |  | 0.97 | 0.59 to 1.60 | 0.908 |
| Main effect | |  |  |  |
| ENI |  | 0.75 | 0.53 to 1.05 | 0.093 |
| Erlotinib |  | 0.74 | 0.52 to 1.04 | 0.082 |

Table S5. Clinical features of EGFR tested group and non-tested group

| **Characteristics** | | **N(%)** |  | **No EGFR** |  | **EGFR** | **p** |
| --- | --- | --- | --- | --- | --- | --- | --- |
|  |  |  |  | **N(%)** |  | **N(%)** |  |
| **Sex** | Male | 288(81.8) |  | 208(79.4) |  | 80(88.9) | 0.044 |
|  | Female | 64(18.2) |  | 54(20.6) |  | 10(11.1) |  |
| **Age (years)** | Median | 61 |  | 61 |  | 59.5 | 0.397 |
|  | Range | 35-70 |  | 35-69 |  | 41-70 |  |
| **Tumor**  **length (cm)** | Median | 5.3 |  | 5.05 |  | 5.2 | 0.556 |
|  | Range | 1.2-15 |  | 1.2-15 |  | 2.2-12 |  |
| **T** | T1 | 11(3.1) |  | 7(2.7) |  | 4(4.4) | 0.767 |
|  | T2 | 47(13.4) |  | 37(14.1) |  | 10(11.1) |  |
|  | T3 | 193(54.8) |  | 143(54.6) |  | 50(55.6) |  |
|  | T4 | 101(28.7) |  | 75(28.6) |  | 26(28.9) |  |
| **N** | N- | 149(42.3) |  | 107(40.8) |  | 42(46.7) | 0.334 |
|  | N+ | 203(57.7) |  | 155(59.2) |  | 48(53.3) |  |
| **ECOG PS** | 0-1 | 218(61.9) |  | 164(62.6) |  | 54(60.0) | 0.662 |
|  | 2 | 134(38.1) |  | 98(37.4) |  | 36(40.0) |  |
| **Tumor**  **location** | Cervical | 21(6.0) |  | 11(4.2) |  | 10(11.1) | 0.000038 |
|  | Upper thoracic | 103(28.7) |  | 75(28.6) |  | 28(31.1) |  |
|  | Middle thoracic | 195(55.4) |  | 160(61.1) |  | 35(38.9) |  |
|  | Lower thoracic | 33(9.4) |  | 16(6.1) |  | 17(18.9) |  |

Table S6. Clinical features of patients with EGFR expression and without EGFR expression

| **Characteristics** | | **N(%)** |  | **EGFR 0/1+** |  | **EGFR 2+/3+** | ***p*** |
| --- | --- | --- | --- | --- | --- | --- | --- |
|  |  |  |  | **N(%)** |  | **N(%)** |  |
| **Sex** | Male | 80(88.9) |  | 36(92.3) |  | 44(86.3) | 0.367 |
|  | Female | 10(11.1) |  | 3(7.7) |  | 7(13.7) |  |
| **Age (years)** | Median | 59.5 |  | 60 |  | 59 | 0.335 |
|  | Range | 41-79 |  | 44-79 |  | 41-70 |  |
| **Tumor**  **length (cm)** | Median | 5.2 |  | 5 |  | 5.5 | 0.734 |
|  | Range | 2.2-12.0 |  | 3-10.1 |  | 2.2-12.0 |  |
| **T** | T1 | 4(4.4) |  | 1(2.6) |  | 3(5.9) | 0.035 |
|  | T2 | 10(11.1) |  | 1(2.6) |  | 9(17.6) |  |
|  | T3 | 50(55.6) |  | 21(53.8) |  | 29(56.9) |  |
|  | T4 | 26(28.9) |  | 16(41) |  | 10(19.6) |  |
| **N** | N- | 42(46.7) |  | 17(43.6) |  | 25(49) | 0.609 |
|  | N+ | 48(53.3) |  | 22(56.4) |  | 26(51) |  |
| **ECOG PS** | 0-1 | 54(60) |  | 24(61.5) |  | 30(58.8) | 0.794 |
|  | 2 | 36(40) |  | 15(38.5) |  | 21(41.2) |  |
| **Tumor**  **location** | Cervical | 10(11.1) |  | 5(12.8) |  | 5(9.8) | 0.462 |
|  | Upper thoracic | 28(31.1) |  | 9(23.1) |  | 19(37.3) |  |
|  | Middle thoracic | 35(38.9) |  | 18(46.2) |  | 17(33.3) |  |
|  | Lower thoracic | 17(18.9) |  | 7(17.9) |  | 10(19.6) |  |
